# Development of auditory and language skills in children using cochlear implants with two signal processing strategies^☆^

**DOI:** 10.1016/j.bjorl.2019.05.006

**Published:** 2019-06-18

**Authors:** Tatiana Mendes de Melo, Elisabete Honda Yamaguti, Adriane Lima Mortari Moret, Orozimbo Alves Costa, Natália Barreto Frederigue Lopes

**Affiliations:** a Universidade de São Paulo (USP), São Paulo, SP, Brazil; b Universidade de São Paulo (USP), Hospital de Reabilitação de Anomalias Craniofaciais, Seção de Implante Coclear, Bauru, SP, Brazil; c Universidade de São Paulo (USP), Departamento de Fonoaudiologia, Bauru, SP, Brazil; d Universidade de São Paulo (USP), Bauru, SP, Brazil

**Keywords:** Hearing loss, Cochlear implant, Child, Language, Auditory perception, Perda auditiva, Implante coclear, Criança, Linguagem, Percepção auditiva

## Abstract

**Introduction:**

The increase in the spectral information offered by the sound processing strategy HiRes 120 has led to great expectations for the pediatric population. Due to a shorter duration of auditory deprivation and higher neural plasticity, children could benefit more substantially from the spectral information of this sound processing strategy.

**Objective:**

To compare auditory and language skills in Brazilian children with cochlear implants using the HiRes and HiRes 120 sound processing strategies.

**Methods:**

Thirty children, aged 1–3 years, with congenital hearing loss, were divided into two groups, according to the signal processing strategy adjusted at the time of the cochlear implant activation. The assessed children were matched according to chronological age and the time of the cochlear implant use. The auditory and language skills were evaluated longitudinally through the Infant-Toddler Meaningful Auditory Integration Scale and Production Infant Scale Evaluation, carried out before surgery, and 3, 6 and 12 months after device implantation. The Mann–Whitney test was applied for the comparison between the two groups with a 5% significance level.

**Results:**

The findings indicated development of hearing and language skills in the first year of cochlear implant use; however, there was no statistically significant difference in the evolution of such skills due to the adjusted processing strategy in the activation of the cochlear implant electrodes.

**Conclusion:**

The development of auditory and language skills in the assessed children was similar during the entire study period, regardless of which signal processing strategy was used.

## Resumo

**Introdução:**

O aumento das informações espectrais proporcionado pela estratégia de processamento de sinal HiRes 120 gera expectativas em relação à população pediátrica, uma vez que devido ao curto período de privação sensorial e da maior plasticidade neural, as crianças poderiam se beneficiar de forma mais substancial das informações espectrais dessa estratégia de processamento de sinal.

**Objetivo:**

Comparar as habilidades auditivas e de linguagem em crianças usuárias de implante coclear, com a estratégia de processamento de sinal HiRes e HiRes 120.

**Método:**

Trinta crianças de um a três anos e com perda auditiva congênita foram divididas em dois grupos, de acordo com a estratégia de processamento de sinal ajustada no momento da ativação do implante coclear. As crianças avaliadas foram pareadas de acordo com a idade cronológica e o tempo de uso do implante coclear. As habilidades auditivas e de linguagem foram avaliadas, longitudinalmente, por meio do *Infant-Toddler Meaningful Auditory Integration Scale* e *Production Infant Scale Evaluation*, antes do implante coclear e com 3, 6 e 12 meses de uso do dispositivo. O teste estatístico de *Mann-Whitney* foi aplicado para comparar os resultados dos grupos, com nível de significância de 5%.

**Resultados:**

Os achados indicaram desenvolvimento das habilidades auditivas e de linguagem no primeiro ano de uso do implante coclear, porém, sem diferença estatisticamente significante na evolução de tais habilidades em função da estratégia de processamento ajustada na ativação dos eletrodos do implante coclear.

**Conclusão:**

O desenvolvimento das habilidades auditivas e de linguagem das crianças avaliadas foi semelhante durante o período do presente estudo, independente da estratégia de processamento de sinal utilizada.

## Introduction

The cochlear implant (CI) represents the most important advance in the treatment of individuals with severe and/or profound bilateral hearing loss who do not benefit from personal sound amplification products (PSAPs). With the technological advancements in this area, it was possible to observe an improvement in the auditory performance among users, not only due to expansion of the indications for cochlear implantation, but also to technological improvements in the implants themselves, in the electrode bundle, microphone technology, signal processing strategies and preprocessing algorithms.^1^

The auditory and language performance of CI users is closely related to factors that include age at surgery,^2^, ^3^ duration of sensory deprivation,^4^, ^5^ effective device use,^6^ the hearing loss etiology,^7^, ^8^, ^9^ the child’s cognitive skills,^10^ family participation in the therapeutic process,^11^ specialized speech-language therapy,^12^ and the optimization of programming parameters,^13^, ^14^, ^15^, ^16^ and other factors.

Among the different programming parameters adjusted at the moment of the mapping, the adopted signal processing strategy defines how the acoustic information will be converted into an electric stimulus. The objective is for the electrical signal to be represented more closely to the acoustic information captured by the CI microphone.

In Brazil, the CI manufacturers Advanced Bionics, Cochlear Corporation, Med-El and Oticon Medical are approved by the National Sanitary Surveillance Agency (ANVISA) to sell their products. The different CI companies advise speech-language pathologists and audiologists of the CI team on the recommended signal processing strategy for each device model.

However, the clinical assessment is extremely important to evaluate the post-CI results with the different signal processing strategies, since sometimes the strategy recommended by the manufacturer does not benefit all CI users in the same way.^15^, ^16^

Advanced Bionics devices were initially marketed in the early 1990s with Clarion 1.0 and 1.2 CI devices. A major technological advance for these devices was the launching of the HiResolution (HiRes) signal processing strategy, which provided greater temporal detail of the acoustic information when compared to the previously used signal processing strategies. This greater detailing provided better results regarding auditory perception, speech intelligibility and oral language in CI users of all age groups, compared to the results of previous generations of signal processing strategies.^17^, ^18^, ^19^, ^20^

In turn, one of the CI limitations is the gap in the detailing of transmitted spectral information, either by the irregular survival of the remaining auditory fibers at the site where the electrode is positioned, or by the limited number of active contacts in the electrode bundle. Due to this limitation, in 2012, the same company implemented a new signal processing strategy, the HiRes 120, which incorporates the detailing of spectral characteristics through the application of the virtual spectral channel technique. Thus, the HiRes 120 allowed an increase in the number of intracochlear stimulation sites and, consequently, provided more spectral information for the CI user, along with the other benefits already provided by the HiRes strategy. Due to the characteristics of this signal processing strategy, the users of these strategies can achieve improvement in the performance of speech perception in noise and musical perception.^21^, ^22^, ^23^

The implementation of HiRes 120 in the pediatric population is more recent than studies carried out in the adult population and has led to high expectations. It is believed that the improvement in spectral information should be more useful in cases of high neural survival, as in children. Because of the short period of sensory deprivation and greater residual neural plasticity, children could more substantially benefit from the spectral information of this signal processing strategy.^24^

Considering the abovementioned issues and the lack of scientific studies analyzing the development of the hearing and language skills of children with CI using the HiRes and HiRes 120 strategies, it is desirable to know which Advanced Bionics signal processing strategy provides better results regarding the development of such skills in children implanted with this device.

## Objective

To longitudinally compare the hearing and language skills in children using cochlear implants in the first year of CI device using the HiRes and HiRes 120 signal processing strategy.

## Methods

Retrospective longitudinal study carried out at the Cochlear Implant Section of Hospital de Reabilitação de Anomalias Craniofaciais of Universidade de São Paulo and approved by the Research Ethics Committee of this Institution, under Official Letter n. 217/2011.

### Sample

Thirty children of both genders with severe and/or profound prelinguistic sensorineural hearing loss, and regularly enrolled in the CI center were implanted with the Advanced Bionics 90K device. All the children participating in the present study were evaluated by the same team of professionals, in the pre- and post-surgical phases. These children were divided into two groups, according to the signal processing strategy used in the first year of CI use, matched according to the chronological age at the time of surgery and time of sensory deprivation:

HiRes Group — 15 children users of CIs, who used the HiRes signal processing strategy since the moment when the electrodes were activated;

HiRes 120 Group — 15 children users of CIs, who used the HiRes 120 signal processing strategy since the moment when the electrodes were activated;

Of the children in the HiRes group, one had hearing impairment due to meningitis, one due to multifactorial issues at birth (preterm birth associated with neonatal ICU admission), three had genetic hearing defects and 10 children had an undetermined etiology. Of the children in the HiRes 120 group, two had hearing impairment due to meningitis, two due to multifactorial issues at birth, two had genetic hearing defects and nine children had an undetermined etiology.

All participants had total electrode insertion and made effective use of the device since its activation (minimum of 8 h daily).

As this was a retrospective study, the Free and Informed Consent form was considered, which was signed by the patients’ parents and/or guardians on the date of the patient’s enrollment in the CI center, authorizing the use of the information described in the medical record for scientific purposes.

At the time of the surgical indication of the CI, the following criteria were adopted for children with prelinguistic hearing loss: age from one to three years; severe and/or profound bilateral sensorineural hearing loss; auditory thresholds with PSAPs >60 dB in speech frequencies; absence of disabilities associated with hearing impairment; auditory rehabilitation carried out at the city of origin; family adequacy and motivation for the CI use.

At the time of the evaluation, all study participants were undergoing speech-language therapy using an aurioral approach, that is, one that emphasizes the stimulation of hearing for the acquisition and development of oral language.

### Tools

To evaluate the hearing abilities, the Infant Toddler: Meaningful Auditory Integration Scale (IT-MAIS) tool, adapted to the Portuguese language was applied,^25^ which aims to evaluate hearing skills in children aged up to four years in situations of daily living. It is a structured questionnaire, presented in the form of a scale, consisting of 10 questions applied to the parents during an interview, assessing three aspects of the auditory skills: relationship with the electronic device (the desire to use it and the ability to detect and identify its malfunction), attention to sound (the child’s spontaneous responses to the auditory stimuli) and ability to attribute meaning to auditory phenomena (the association of sound to its meaning). The answers can vary on a five-point scale, which shows the percentage of children that demonstrates the assessed auditory skills, with a score of zero to four, that is, 0 = never, the child has 0% of the assessed hearing skills; 1 = rarely, the child demonstrates the assessed skill 25% of the time; 2 = occasionally, the child demonstrates the assessed skill 50% of the time; 3 = often, the child demonstrates the assessed skill 75% of the time; and 4 = always, the child demonstrates the assessed skill 100% of the time. The maximum score that can be reached in the questionnaire is 40 points or 100%.

The Production Infant Scale Evaluation (PRISE)^26^ questionnaire was applied to assess language skills and aims to access the prelinguistic production of children in situations of daily living. It consists of 11 questions, applied to parents in the form of an interview. Response options vary on a five-point scale, which show the percentage demonstrated by the child regarding her pre-linguistic ability, with scores varying from 0 to 4, that is, 0 = never, the children demonstrates 0% of the assessed pre-linguistic skill; 1 = rarely, the child demonstrates the assessed pre-linguistic skill 25% of the time; 2 = occasionally, the child demonstrates the assessed skill 50% of the time; 3 = often, the child demonstrates the assessed skill 75% of the time and 4 = always, the child demonstrates the assessed skill 100% of the time. The maximum score that can be reached in the questionnaire is 44 points or 100%.

### Procedures

All children enrolled in the CI Center were regularly followed every three months in the first year of CI use. Several procedures were applied throughout the treatment routine, including the application of the IT-MAIS and PRISE questionnaires. Thus, for the present study, the results of the previously mentioned questionnaires applied in the preoperative evaluation stage (pre-CI) and during the follow-up consultations at 3, 6 and 12 months of Ci use were collected from the participants’ files.

### Analysis of results

The analysis of the results was carried out through descriptive statistics of the two study groups. Subsequently, the inferential statistical analysis was performed using the Mann–Whitney test, to compare the results of the IT-MAIS and PRISE questionnaires, at the different moments of assessment. The STATA software package, version 9.0, was used for the statistical analysis, and the significance level was set at 5%.

## Results

The demographic characteristics of the study participants are shown in Table 1. There was no difference between the groups regarding age and time of sensory deprivation.

**Table 1:** Demographic characteristics of the assessed children.

	Mean	Standard deviation	Minimum	Maximum	*p*
Age (months)					1.00
HiRes group	26.2	8.3	14	41	
HiRes 120 group	25.5	8.1	12	40	

Time of deprivation (months)					0.91
HiRes group	27.0	8.3	15	42	
HiRes 120 group	26.6	8.0	13	41	

**Figure 1:**
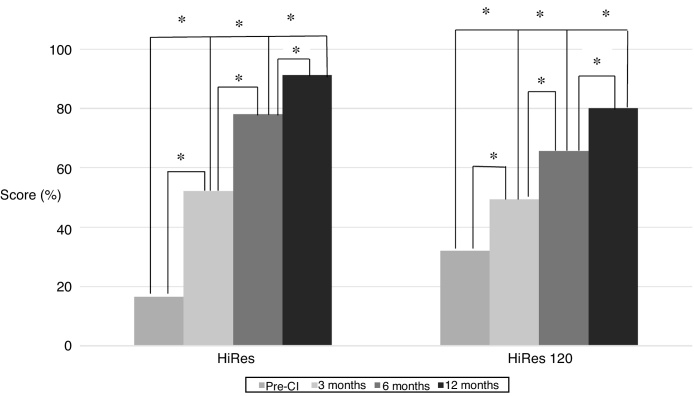
Performance of the hearing skills in the assessed children using the IT-MAIS questionnaire, in the pre-CI assessment and after 3, 6 and 12 months of CI use. The asterisk indicates a statistically significant difference between the groups at the different moments of the study (*p* < 0.05).

**Figure 2:**
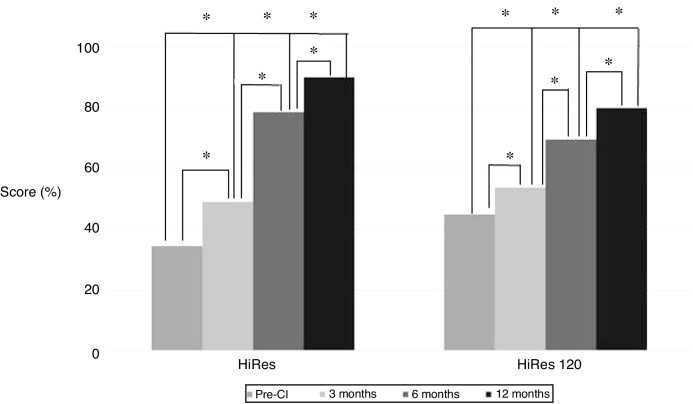
Performance of the language skills of the assessed children, assessed through the PRISE questionnaire, in the pre-CI evaluation and after 3, 6 and 12 months of CI use. The asterisk indicates a statistically significant difference between the groups at the different moments of the study (*p* < 0.05).

The results obtained after the application of the IT-MAIS and PRISE questionnaires in both groups during the study period are shown in Figure 1, Figure 2. The findings disclose that, regardless of the signal processing strategy used, development of auditory and language skills was observed, with a statistically significant difference after the first year of CI use.

**Figure 3:**
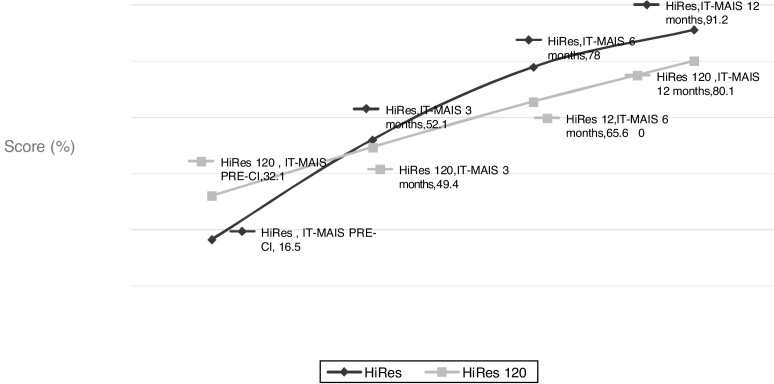
Performance of hearing skills, evaluated through the IT-MAIS questionnaire, at the different moments evaluated by the study, according to the programmed signal processing strategy at the CI activation.

**Figure 4:**
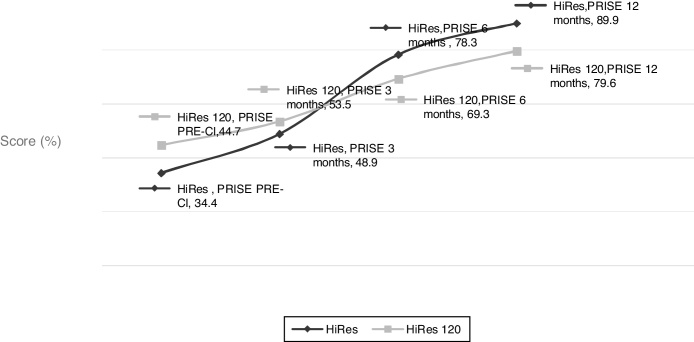
Performance of language skills, evaluated through the PRISE questionnaire, in the different moments evaluated by the study, according to the programmed signal processing strategy at the CI activation.

When comparing the development of the hearing and language skills in the group of activated children by the signal processing strategy, it was not possible to observe any statistically significant differences between the groups at any of the assessed moments, although there was a trend of better results in the group who used the HiRes strategy, from the third and sixth month of the CI use (Figure 3, Figure 4), for the auditory and language skills, respectively.

## Discussion

The aim of the present study was to longitudinally compare the hearing and language skills in children using cochlear implants in the first year of device use, while using the HiRes and HiRes 120 signal processing strategy, aiming to provide a better basis for professionals in the area to optimize the device programming parameters.

The findings show a marked development of auditory and language skills in the first year of CI use, with no difference regarding the evolution of these skills related to the processing strategy adjusted after the activation of the CI electrodes.

The electrical stimulation provided by the effective use of the CI, associated to auditory rehabilitation, allows the development of the central auditory pathways and, when surgery is performed early, it allows this development to occur concomitantly with the critical maturation period of this sensory system, providing better opportunities for acquiring auditory and language skills.^2^, ^3^, ^6^, ^12^^,^^27^, ^28^

The IT-MAIS questionnaire has been widely used, both nationally and internationally, to monitor the development of auditory skills, especially in the first years of CI use, through the observation of the auditory behavior. The findings have shown the evolution of such skills during the first year of device use, in a statistically significant way, in both assessed groups (Fig. 1).

It is also possible to observe that, even before the CI activation (pre-CI evaluation), the assessed children already showed an initial development of auditory skills. The evaluation of the benefit provided by the PSAP is essential for the CI indication process and, with the adaptation of this device, it is possible that the amplified acoustic signal allows the initial development of the auditory behavior.

In turn, the PRISE scale, used in this study to assess language skills in the first year of CI use, evaluates the prelinguistic development and is extremely important as an indicator of language acquisition and development in the implanted children.

According to the literature,^26^ children with severe and/or profound hearing loss show a score below 50% on this scale, regardless of the child’s chronological age at the pre-surgical time, corroborating the results shown by the participants of the present study before the CI surgery. However, there is an increase in vocalizations (undifferentiated and differentiated) after the CI, regardless of the signal processing strategy used, which can be attributed to the auditory feedback provided by the device (Figure 2, Figure 4).

When comparing the hearing development in the first year of the CI use in the two assessed groups, it was evident that there was a tendency of the children using the HiRes strategy to exhibit better performance of the skills assessed from the third month onward; however, this difference was not statistically significant in any of the assessed intervals (Fig. 3). Regarding language skills, this trend of better results with the HiRes signal processing strategy was present as of the 6th month of device use, but once again, the difference in language performance in the two assessed groups is not statistically significant at any time throughout the study (Fig. 4).

The literature to date shows that the HiRes 120 strategy provides better results in tests of speech perception in noise and in musical appreciation for users of this strategy.^22^, ^24^ Although the HiRes 120 signal processing strategy may provide greater spectral detail of the acoustic signal, with the increase in the number of intracochlear stimulation sites, associated with the already existing higher temporal resolution in the HiRes strategy, this characteristic does not seem to have contributed to a faster rhythm of auditory and language skill development in the first year of CI use.

The present study is a precursor in the investigation of auditory and language development in the first year of CI use, due to the signal processing strategy adjusted at the device programming, thus helping the speech-language pathologists and audiologists that work with mapping to understand the importance of the professional’s clinical assessment during the device use.

The study limitations should also be considered. Despite the tendency among the users of the HiRes strategy to achieve better performance of the assessed skills, a larger group of evaluated children could indicate a significant difference between the groups. In this context, it is important to continue investigations in this area, since, in general, CI manufacturers recommend certain adjustments to the device programming to their users; however, not all patients might benefit from the recommended parameters in the same way.

## Conclusion

The development of hearing and language skills of the assessed children was similar during the present study period, regardless of the signal processing strategy used.

## Conflicts of interest

The authors declare no conflicts of interest.
